# Clinical endocrinological evaluation of the gonadal axis (testosterone, LH and FSH) in prostate cancer patients switched from a GnRH antagonist to a LHRH agonist

**DOI:** 10.1186/s12610-015-0023-2

**Published:** 2015-07-03

**Authors:** Yoshiyuki Miyazawa, Haruo Kato, Seiji Arai, Yosuke Furuya, Yoshitaka Sekine, Masashi Nomura, Hidekazu Koike, Hiroshi Matsui, Yasuhiro Shibata, Kazuto Ito, Kazuhiro Suzuki

**Affiliations:** Department of Urology, Gunma University Graduate School of Medicine, 3-39-22 Showa-machi, Maebashi 371-8511 Gunma, Japan

**Keywords:** Prostate cancer, GnRH-antagonist, LHRH-agonist, Testosterone

## Abstract

**Objectives:**

To investigate the levels of testosterone, luteinizing hormone (LH), follicle-stimulating hormone (FSH), and prostate-specific antigen (PSA) in prostate cancer patients before and after the switch from degarelix to leuprolide treatments.

**Methods:**

We enrolled 40 treatment-naïve prostate cancer patients who were treated initially with degarelix and were later switched to leuprolide. The subjects were divided into three groups depending on when they were switched to leuprolide: the 3-month group (3m; switched after 84 days, *n*=10), the 2-month group (2m; 56 days, *n*=10), and the 1-month group (1m; 28 days, *n*=20). Patient symptoms and hormone levels were measured after switching therapy. The castration level was defined as a serum testosterone level ≤50 ng/dl.

**Results:**

Thirty-nine subjects (97.5%) achieved castration levels of testosterone (11±5.8 ng/dl) 2 weeks after degarelix was first administered, and the characteristics of these patients were investigated. Testosterone levels increased and exceeded the castration level in one subject each of the 3m (142 ng/dl), 2m (72 ng/dl), and 1m groups (63 ng/dl). All subjects achieved the castration level by day 5. In contrast to testosterone levels, the LH and FSH surge on day 2 was significantly higher in the 1m group than in the other groups. The clinical symptoms were not exacerbated before or after switching in any patients.

**Conclusions:**

A testosterone surge was observed in 8.3 % of the study patients; however, it was very short-lived and mild. LH and FSH levels were significantly higher 1 month after administration compared with 2 or 3 months after degarelix administration.

## Introduction

Prostate cancer is the most common cancer in males in Western countries, and its incidence has increased in Japan [1]. Prostate cancer is dependent on androgens; therefore, patients are often treated with androgen deprivation therapy (ADT) [2]. Luteinizing hormone-releasing hormone (LHRH) agonists are the most prevalent form of ADT. The main concern with the use of LHRH agonists for ADT is the clinical worsening of symptoms due to a testosterone surge upon initiation of LHRH agonist treatment. Therefore, antiandrogens are often used to reduce the risk of flare up. However, potential adverse drug reactions and the added cost of antiandrogens must be considered.

The gonadotropin releasing hormone (GnRH) antagonist degarelix is a newly discovered agent that blocks GnRH receptors immediately and testosterone production rapidly, preventing a surge. However, degarelix, the GnRH antagonist currently available in Japan, has therapeutic effects that last for only 1 month, and thus, patients must commute to the hospital once a month to receive subcutaneous injections. In contrast, the LHRH agonists leuprolide and goselerin are available as 1 and 3 month formulations, respectively. The interval between hospital visits using 3-month formulations helps reduce the number of hospital visits, as well as the number of subcutaneous injections. Therefore, when ADT treatment is initiated, patients are often switched to a LHRH agonist empirically after the state of castration is reached using degarelix, thereby precluding the need for an antiandrogen. After switching, the patients gain the benefit of longer intervals between hospital visits.

Garnick et al. firstly reported switching antagonist to agonists. They demonstrated the endocrinological and biochemical efficacy of initiating treatment with the GnRH antagonist abarelix followed by the administration of LHRH agonists [3]. Zuckerman et al. performed a prospective evaluation of the testosterone surge when treatment was switched from 3-month depot injections of degarelix to leuprolide [4]. They reported that the fluctuations in serum testosterone were mild and short-lived, and that there were no symptomatic patients after switching [4]. Based on these reports, we conducted the current study to investigate the trends in hormone levels before and after prostate cancer patients were switched from degarelix to leuprolide. We also assessed the changes in hormone levels during different periods of degarelix administration (1, 2, and 3 months). This is the first prospective evaluation of hormone levels in the switch from degarelix to leuprolide in Japanese patients.

## Methods

We conducted an investigator-initiated, prospective, single-arm, open-label trial. The primary objective of the study was to evaluate the surge in testosterone, luteinizing hormone (LH), follicle-stimulating hormone (FSH), and prostate-specific antigen (PSA) and to determine whether symptoms worsened during the switch from degarelix to leuprolide. We defined “testosterone surge” as an increase in testosterone that is an obligate increase whether an antiandrogen is used or not, and “clinical flare” as worsening of symptoms due to the increase in testosterone levels. The subjects included 40 treatment-naïve prostate cancer patients (mean age 70.6 ± 7.0 years, range 55−85, mean of body weight 64.2 ± 7.87 kg, range 49−80, mean of Body Mass Index 23.1 ± 2.65, range 16.6−27.7, data are presented as mean ± Standard Deviation, S.D.) who were treated initially with degarelix hormone therapy and later switched to leuprolide. The characteristics of patients are shown in Table 1. Because of concern about the occurrence of testosterone flare, which worsens pain upon bone metastasis, we selected only patients who had no such metastasis. 24 patients (mean age 67.6 years) received radiation therapy with hormonal therapy. 16 patients (mean of age 75.7) were treated by continuous androgen deprivation therapy for their preference. The subjects were divided into three groups depending on when they were switched to 3.75 mg leuprolide. The 3-month (3 m) group was given 240 mg degarelix *via* two 120-mg deep subcutaneous injections, to initiate ADT, at day -84, followed by two 80-mg maintenance doses (*n* = 10). The 2-month (2 m) group was treated with 240 mg degarelix at day -56 *via* two 120-mg deep subcutaneous injections, to initiate ADT, followed by a single 80-mg maintenance dose (*n =* 10). Finally, patients in the 1-month (1 m) group were treated with 240 mg degarelix *via* two 120-mg deep subcutaneous injections to initiate ADT at day -28, with no maintenance dose (*n =* 20). The day on which the medications were switched was recorded as day 0. The symptoms were confirmed, and hormone levels were measured on days 0, 1, 2, 5, 7, and 28. No patient received any antiandrogen. Serum LH and FSH were measured using a chemiluminescent immunoassay (CLIA). PSA was measured using a chemiluminescent enzyme immunoassay (CLEIA). Testosterone in all samples was measured with an electrochemiluminescence Immunoassay (ECLIA). It has been reported that the correlation coefficients for automated immunoassays are relatively poor when testosterone concentrations are low (<4.0 nmol/L) [5], so we also measured serum testosterone on days 0, 1, 2, 5 and 7 *via* liquid chromatography-tandem mass spectrometry (LC-MS/MS). The castration level was defined as a serum testosterone level ≤50 ng/dl. This study was conducted with approval from the Gunma University Clinical Research Ethics Committee. Friedman and Wilcoxon Signed-Rank tests and one-factor ANOVA were used to compare PSA and hormonal parameters among the groups. Multivariate analyses were performed using a linear regression model, and *p* < 0.05 was considered indicative of statistical significance. All statistical analyses were performed using commercially available statistical software (SPSS ver. 21.0 IBM, Chicago, IL, USA).

**Table 1:** Clinical characteristics of the patients

	All patients	3 m group	2 m group	1 m group	*p*-value
*n*	40	10	10	20	
Age	70.6 ± 7.0	73.4 ± 7.9	72.1 ± 6.9	68.5 ± 6.2	0.147
BW (kg)	64.2 ± 7.9	61.7 ± 6.9	62.0 ± 8.9	66.1 ± 6.8	0.289
Height (cm)	166.6 ± 5.1	166.9 ± 3.4	164.5 ± 6.2	165.9 ± 5.3	0.166
BMI	23.1 ± 2.6	22.1 ± 2.3	22.9 ± 2.9	24.0 ± 2.1	0.295
PSA	12.0 ± 9.9	8.91 ± 7.10	17.6 ± 14.9	10.65 ± 6.84	0.177
GS; *n* (%)					
6	5 (12)		2 (20)	3 (15)	
7	22 (55)	7 (70)	4 (40)	11 (55)	
8	8 (20)	3 (30)	2 (20)	3 (15)	
9	5 (13)		2 (20)	3 (15)	
TNM classification; *n* (%)					
T1cN0M0	5 (13)		2 (20)	3 (15)	
T2N0M0	22 (55)	8 (80)	4 (40)	10 (50)	
T3N0M0	13 (33)	2 (20)	4 (40)	7 (35)	
D’Amico risk classification; *n* (%)					
low	2 (7)		1 (10)	1 (5)	
intermediate	12 (40)	3 (30)	2 (20)	7 (35)	
poor	16 (53)	7 (70)	7 (70)	12 (60)	

Data are presented as means ± SD unless indicated otherwise

*BW* body weight; *BMI* Body Mass Index; *PSA* prostatic specific antigen; *GS* Gleason score; *SD* standard deviation

## Results

A total of 40 patients aged 55–85 years (70.6 ± 7.0, mean ± S.D.) were enrolled in the study between July and October of 2013. The baseline patient characteristics are shown in Table 1.

### Testosterone determined by ECLIA

The mean testosterone level before initiating ADT using degarelix was 470.4 ± 161.1 (mean ± S.D.) ng/dl. One patient in the 1 m group was excluded from the analysis because his testosterone did not reach castration level (166 ng/dl) before switching to leuprolide. After administering a 3.75-mg dose of leuprolide, he reached castration levels successfully (6.0 ng/dl). Ultimately, 39 patients who reach castration level (mean of testosterone 7.5 ± 5.8 ng/dl, mean ± S.D.) before switching to leuprolide from degarelix were included in the analysis. The data are shown in Table 2. A testosterone surge of up to 50 ng/dl was observed in three cases (7.7 %) (59.0 ng/dl in one patient, No.30 in the 1 m group, 69.0 ng/dl in one patient, No.18 in the 2 m group, and 161.0 ng/dl in one patient, No.10 in the 3 m group).

**Table 2:** Serum testosterone on each days after switching to lueprolide

		Day 0	Day 1	Day 2	Day 5	Day 7
ECLIA	Total (*n* = 39)	7.5 ± 5.8	19.7 ± 27.5	17.4 ± 18.2	7.0 ± 4.8	6.7 ± 4.6
	Surge (-)					
	Total (*n* = 36)	7.6 ± 6.0	13.5 ± 10.6	14.3 ± 11.2	7.1 ± 4.8	6.8 ± 4.7
	3 m group (*n* = 9)	8.2 ± 6.8	11.9 ± 10.6	11.9 ± 11.4	7.9 ± 5.7	6.9 ± 5.0
	2 m group (*n* = 9)	6.0 ± 5.6	10.6 ± 11.9	9.0 ± 8.0	6.0 ± 5.7	6.3 ± 5.6
	1 m group (*n* = 19)	8.2 ± 5.9	15.7 ± 5.9	18.2 ± 11.5	7.2 ± 4.0	7.1 ± 4.1
	Surge (+)					
	No.10 (3 m)	10.0	161.0	88.0	3.0	3.0
	No.18 (2 m)	3.0	63.0	69.0	3.0	3.0
	No.30 (1 m)	3.0	59.0	5.0	12.0	9.0
LC-MS/MS	Total (*n* = 36)	8.6 ± 4.4	24.1 ± 25.3	22.6 ± 20.6	8.6 ± 2.7	8.0 ± 3.0
	Surge (-)					
	Total (*n* = 33)	7.9 ± 3.0	17.9 ± 10.3	19.1 ± 13.4	8.4 ± 2.6	7.6 ± 2.6
	3 m group (*n* = 9)	8.6 ± 3.3	15.7 ± 12.3	15.4 ± 11.5	8.4 ± 3.4	8.0 ± 3.1
	2 m group (*n* = 8)	6.4 ± 2.6	14.9 ± 12.5	11.1 ± 11.1	6.8 ± 2.7	6.6 ± 3.2
	1 m group (*n* = 16)	8.3 ± 2.9	20.8 ± 7.4	24.4 ± 13.7	9.2 ± 1.6	7.9 ± 1.9
	Surge (+)					
	No.10 (3 m)	28.0	142.0	93.0	12.0	16.0
	No.18 (2 m)	12.0	72.0	80.0	9.0	12.0
	No.30 (1 m)	7.0	63.0	7.0	12.0	9.0

Data are presented as means ± SD unless indicated otherwise

*ECLIA* electrochemiluminescence Immunoassay; *LC-MS/MS* liquid chromatography-tandem mass spectrometry; surge (-) means a testosterone surge of down to 50 ng/dl was observed, surge (+) means a testosterone surge of up to 50 ng/dl was observed

### Testosterone determined by LC-MS/MS

**Fig. 1:**
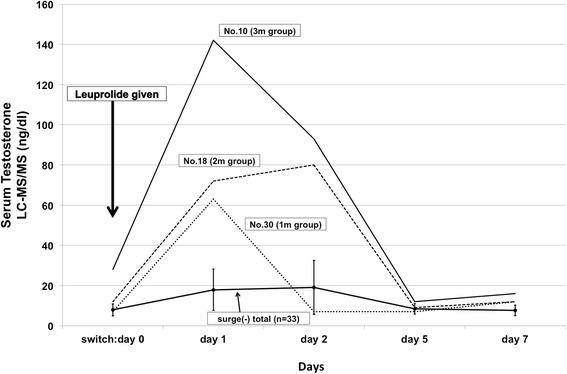
Serum testosterone levels (LC-MS/MS) of the study patients at various time points. After the administration of leuprolide, a testosterone surge of up to 50 ng/dl was observed in same three cases (63 ng/dl: No.30 in the 1 m group, 80 ng/dl: No.18 in the 2 m group, and 142 ng/dl: No.10 in the 3 m group). “surge (-) total (*n* = 33)” shows mean of serum testosterone in 33 patients who has no testosterone surge of up to 50 ng/dl. Error bar shows S.D

We measured serum testosterone on days 0, 1, 2, 5, and 7 *via* LC-MS/MS because it has been reported that the correlations between the results of automated immunoassays are poor when testosterone concentrations are low (<4.0 nmol/L) [5]. We were not able to evaluate testosterone levels in three patients on the day of the switch from degarelix (1 patient in 2 m group, 2 patients in 1 m group) because no serum remained after ECLIA assay. We evaluate testosterone by LC-MS/MS in 36 patients. The data are shown in Table 2. In 36 patients, mean testosterone level was 8.6 ± 4.4 ng/dl on day of switching to leuprolide from degarelix as day 0, 24.1 ± 25.3 ng/dl on day 1, 22.6 ± 20.6 ng/dl on day 2, 8.6 ± 2.7 ng/dl on day 5, 8.0 ± 3.0 ng/dl on day 7 (mean ± S.D.). Similar with the results found with the ECLIA, after the administration of leuprolide, a testosterone surge of up to 50 ng/dl was observed in the same three cases (8.3 %) (63 ng/dl in one patient, No.30 in the 1 m group, 80 ng/dl in one patient, No.18 in the 2 m group, and 142 ng/dl in one patient, No.10 in the 3 m group). These three patients returned to castration level within 5 days of switching, and all patients maintained testosterone below the castration level until the end of the observation period (Fig. 1). A testosterone surge was observed in 8.3 % of the study 36 patients by LC-MS/MS. In 33 patients who had no testosterone surge of up to 50 ng/dl, mean testosterone level was 7.9 ± 3.0 ng/dl on day 0, 17.9 ± 10.3 ng/dl on day 1, 19.1 ± 13.4 ng/dl on day 2, 8.4 ± 2.6 ng/dl on day 5, 7.6 ± 2.6 ng/dl on day 7 (mean ± S.D.). There was no significant difference in the width or frequency of the testosterone surge among the three groups.

### PSA

PSA was reduced in all groups after the administration of degarelix (mean PSA ± S.D. before *vs.* after: 3 m group, 9.88 ± 6.97 *vs.* 0.63 ± 0.61 ng/ml; 2 m group, 17.8 ± 14.8 *vs.* 2.05 ± 4.30 ng/ml; 1 m group, 11.8 ± 7.30 *vs.* 3.91 ± 4.32 ng/ml). There were no significant elevations of PSA levels in any of the groups after switching to leuprolide (Table 3).

**Table 3:** Serum PSA on each days after switching to lueprolide

	Day 0	Day 1	Day 2	Day 5	Day 7
total (*n* = 39)	2.61 ± 3.86	2.47 ± 3.66	2.43 ± 3.75	1.95 ± 2.43	1.92 ± 2.63
3 m group (*n* = 10)	0.628 ± 0.611	0.614 ± 0.592	0.631 ± 0.625	0.665 ± 0.621	0.655 ± 0.614
2 m group (*n* = 10)	2.05 ± 4.30	1.94 ± 4.04	2.05 ± 4.31	2.01 ± 3.99	2.13 ± 4.44
1 m group (*n* = 19)	3.88 ± 4.21	3.66 ± 4.02	3.52 ± 4.10	2.58 ± 1.71	2.53 ± 1.68

Data are presented as means ± SD (ng/ml) unless indicated otherwise

*PSA* Prostate Specific Antigen

### LH and FSH

**Fig. 2:**
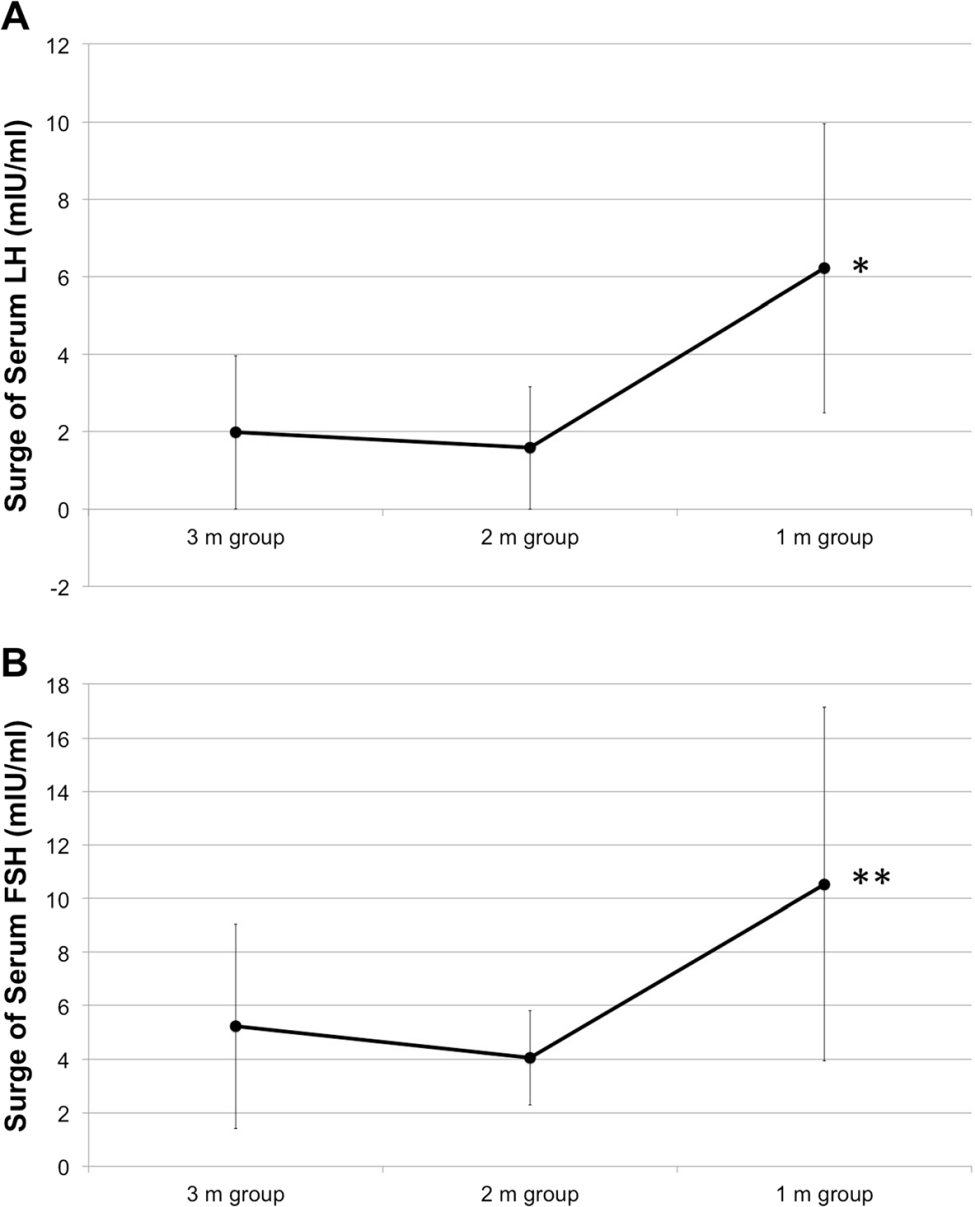
Mean serum LH and FSH surge in the study patients on day 2. **a** LH levels were significantly higher in the 1 m group than the other two groups (*; *vs.* 3 m group, *p =* 0.023; *vs.* 2 m group, *p* = 0.009; Fig. 2a). **b** The FSH surge was higher in the 1 m group than in the other two groups (**; *vs.*3 m group, *p =* 0.019; 2 m group, *p* = 0.001; Fig. 2b). Error bar shows S.D

In contrast, there were significant differences in the median LH surge after switching. The mean of LH surge on day 2 was 1.97 ± 1.97 mIU/ml in the 3 m group, 1.57 ± 1.58 mIU/ml in the 2 m group, and 6.21 ± 3.73 mIU/ml in the 1 m group (mean ± S.D.) . LH levels were significantly higher in the 1 m group than the other two groups (*vs.* 3 m group, *p =* 0.023; *vs.* 2 m group, *p* = 0.009; Fig. 2a). Similarly, there was also an FSH surge on day 2. The mean of FSH surge was 5.24 ± 3.81 mIU/ml in the 3 m group, 4.05 ± 1.77 mIU/ml in the 2 m group, and 10.53 ± 6.59 mIU/ml in the 1 m group (mean ± S.D.). The FSH surge was higher in the 1 m group than in the other two groups (*vs.*3 m group, *p =* 0.019; 2 m group, *p* = 0.001; Fig. 2b).

### Predictive factors of testosterone surge

We performed univariate and multivariate logistic analyses in order to predict which patients might more likely to experience a testosterone surge, measured *via* LC-MS/MS, during the switch from degarelix to leuprolide. The results of multivariate analysis (linear analysis) are shown in Table 4. The period of degarelix administration and LH levels at the time of switching to leuprolide were significantly related to a testosterone surge with the switch to leuprolide. The testosterone surge could be predicted using the following formula: predicted testosterone surge (ng/dl) = 100 × { 0.196 + (0.445 × serum LH on switching [mIU/ml]) + (−0.091 × period of administration of degarelix [months]) }. The testosterone surge decreased with an increasing period of administration of degarelix. Conversely, the testosterone surge increased as the LH level upon switching to leuprolide increased. No patients experienced symptoms associated with a testosterone surge during the observation period. In addition, we found no significant changes in PSA level in any patient after switching to leuprolide.

**Table 4:** Multivariate analysis for predicting testosterone surge (LC-MS/MS)

	Univariate analysis	Multivariate analysis (linear analysis)			
Variables	*P* value	Partial regression coefficient	95% CI lower	95% CI upper	*P* value
Age	0.140				
Period of degarelix administration	0.004	−0.091	−0.150	−0.032	0.004
GS	0.354				
Stage	0.241				
D’Amico classification	0.149				
Serum T (before ADT)	0.688				
Serum T (after switching therapy)	0.948				
Serum LH (before ADT)	0.820				
Serum LH (after switching therapy)	<0.001	0.445	0.339	0.551	<0.001
Serum FSH (before therapy)	0.747				
Serum FSH (after switching therapy)	0.406				

*GS* Gleason score; *ADT* androgen deprivation therapy; *T* testosterone; *LH* luteinizing hormone; *FSH* follicle-stimulating hormone; *CI* confidence interval

## Discussion

ADT has been a long-term treatment for prostate cancer patients since it was first reported by Huggins et al. [6]. It is used as the first-line treatment for patients with metastatic prostate cancer. LHRH agonists are used for castration, and their effectiveness is equivalent to that of surgical castration [7]. However, the testosterone surge that occurs during the early stage of treatment remains problematic because it might exacerbate the disease state temporarily [8]. Reports have suggested that patients with bone metastasis might experience a worsening of bone pain, and that a worsening of symptoms associated with spinal cord compression might be observed in patients with vertebral metastasis. A review of 765 patients in 9 series found 10.9 % who suffered disease flare and 15 who died during disease flare. (in Thompson et al.) [9]. To manage this testosterone surge, oral antiandrogens such as bicalutamide and flutamide are generally administered before initiating treatment with LH-RH agonists [10–12]. However, reports have suggested that it is not possible to prevent the testosterone surge completely, even when antiandrogens are used before LHRH agonists. Klotz et al. reported the efficacy and safety of degarelix in a phase III study in 2008. A total of 201 patients were treated with 7.5 mg leuprolide per month, and 23 patients (11 %) received concomitant bicalutamide for flare protection at the start of treatment. The 144 patients (81 %) without bicalutamide experienced a testosterone surge (an increase of >15 % from baseline). Despite of receiving bicalutamide, 17 of 23 (74 %) patients experienced a similar testosterone surge [13].

GnRH antagonists do not cause a testosterone surge, and they can produce a state of castration more rapidly than LHRH agonists. Studies assessing the therapeutic effects of GnRH antagonists in patients with metastasis demonstrated that the period prior to PSA re-elevation was prolonged, and the effects long-term [14]. Two reports described switching prostate cancer patients from an antagonist to an agonist. First, Garnick et al. demonstrated the safety and endocrinological and biochemical efficacy of initiating treatment with the LH-RH antagonist abarelix followed by the administration of agonists. A total of 176 patients received ADT with abarelix for 12 weeks, followed by maintenance ADT with a LH-RH agonist for 8 weeks. It was reported that 93.8 % of patients attained castration levels of testosterone (<50 mg/dL) after initiation of abarelix therapy, and that 91 % of patients remained at such levels after switching to the agonist. In addition, the mean testosterone levels of the 176 patients increased by a mean of 19.6 ng/dl (range, 17.7–37.3 ng/dl) the day after switching, and no symptomatic progression occurred as a result of switching therapy [3]. A subsequent study by Zuckerman et al. performed a prospective evaluation of testosterone fluctuations associated with the switch from degarelix to leuprolide in 45 patients. They observed a rise in mean testosterone from a nadir of 16.5 ng/dl to a peak of 25.8 ng/dl (*p* = 0.0005). Four patients (8.9 %) experienced a testosterone surge with a mean peak serum level of 80.7 ng/dl the day of switching; all four patients returned to castrate levels within 7 days of switching, and no patients experienced a symptomatic flare [4].

In the current study, we observed a testosterone surge of 50 ng/dl or greater in one of 10 subjects who received degarelix every 3 months (patient no. 10), one of 9 subjects who received degarelix every 2 months (patient no. 18), and one of 17 who received degarelix once a month (patient no. 30). All patients who experienced a testosterone surge returned to the castrate state within 3 days of switching. No significant differences were observed regarding the severity or frequency of the testosterone surge among groups. In addition, no patient experienced symptomatic exacerbations related to the testosterone surge because no patient had bone metastasis, and no elevation in PSA level was noted after switching. Therefore, although surges were observed at the time of switching, none reached a level that was considered clinically problematic. Therefore, it seems that transition of therapy from degarelix to leuprolide is safe after any duration of treatment with degarelix (3 months, 2 months, or 1 month).

Interesting results were observed regarding the LH and FSH dynamics during degarelix administration and at the time of switching. The surges of LH and FSH were larger in the 1-month group, which suggests that the suppression of the pituitary gland was weaker. In addition, the surge at the time of agonist use was not stronger in individuals receiving the 240 mg dose of degarelix 2 or 3 months before the switch to leuprolide compared to those who received the 240 mg dose 1 month before the switch. Although there were differences in the LH and FSH surges according to the period of degarelix administration, there were no significant differences in the mean of testosterone surge among groups, which might have been caused by a suppression of the testicular response. When we focused on individual cases we found that a small testosterone surge was observed in a few patients and that it did not induce a change in PSA levels. The testosterone surges observed in the present study were mainly affected by LH levels on switching to leuprolide and period of administration of degarelix. Although it is possible that a testosterone surge might still occur because of decreased serum concentrations of the antagonist immediately after switching from a LHRH agonist, Zuckerman et al. reported that no testosterone surge was evident immediately after switching, according to testosterone level measurements in samples taken 1, 2, 3, and 4 weeks after switching to the agonist [4].

In the current study, treatment was initiated using a GnRH antagonist, and subjects were then switched to an LHRH agonist. Although testosterone surges were observed in some subjects, they were small and none reached a level that was clinically problematic; this suggests that this treatment is safe. However, the current study did not include subjects with bone metastasis. In addition, patients with multiple metastatic lesions at the time of the initial examination and malignant tumors with a high Gleason score might benefit more from the use of an antagonist than an agonist during long-term ADT therapy. In Japan, the combined androgen blockade using LHRH agonist and antiandrogens is popular in both non-metastatic and metastatic stages of prostate cancer [15, 16]. Based on the present findings, switching from degarelix to leuprolide appears to be a reasonable therapeutic option in prostate cancer patients without metastatic extension, since it does not result in a disease flare-up, and that it does not require an additional treatment with anti-androgens.

## Conclusion

GnRH antagonists such as degarelix can rapidly produce a state of castration. The mean level of testosterone in patients of our study before initiating ADT using degarelix was 470.4 ± 161.1 ng/dL, compared to 8.5 ± 4.4 ng/dL before switching to leuprolide. A testosterone surge was observed in 8.3 % of the study patients; however, it was very short-lived and mild. There was no significant change in PSA level in any group after switching to leuprolide. No patients experienced symptoms associated with a testosterone surge during the observation period. In contrast, we found significant differences in the median LH and FSH surges after switching. LH and FSH surge levels were significantly higher 1 month after degarelix administration compared to 2 or 3 months thereafter. Based on the present findings, switching from degarelix to leuprolide appears to be a reasonable therapeutic option in prostate cancer patients without metastatic extension, since it does not result in a disease flare-up, and that it does not require an additional treatment with anti-androgens.
